# Are Protein Domains Modules of Lateral Genetic Transfer?

**DOI:** 10.1371/journal.pone.0004524

**Published:** 2009-02-20

**Authors:** Cheong Xin Chan, Aaron E. Darling, Robert G. Beiko, Mark A. Ragan

**Affiliations:** 1 The University of Queensland, Institute for Molecular Biosciences, and ARC Centre of Excellence in Bioinformatics, Brisbane, Australia; 2 Faculty of Computer Science, Dalhousie University, Halifax, Nova Scotia, Canada; Georgia Institute of Technology, United States of America

## Abstract

**Background:**

In prokaryotes and some eukaryotes, genetic material can be transferred laterally among unrelated lineages and recombined into new host genomes, providing metabolic and physiological novelty. Although the process is usually framed in terms of gene sharing (*e.g.* lateral gene transfer, LGT), there is little reason to imagine that the units of transfer and recombination correspond to entire, intact genes. Proteins often consist of one or more spatially compact structural regions (domains) which may fold autonomously and which, singly or in combination, confer the protein's specific functions. As LGT is frequent in strongly selective environments and natural selection is based on function, we hypothesized that domains might also serve as modules of genetic transfer, *i.e.* that regions of DNA that are transferred and recombined between lineages might encode intact structural domains of proteins.

**Methodology/Principal Findings:**

We selected 1,462 orthologous gene sets representing 144 prokaryotic genomes, and applied a rigorous two-stage approach to identify recombination breakpoints within these sequences. Recombination breakpoints are very significantly over-represented in gene sets within which protein domain-encoding regions have been annotated. Within these gene sets, breakpoints significantly avoid the domain-encoding regions (*domons*), except where these regions constitute most of the sequence length. Recombination breakpoints that fall within longer domons are distributed uniformly at random, but those that fall within shorter domons may show a slight tendency to avoid the domon midpoint. As we find no evidence for differential selection against nucleotide substitutions following the recombination event, any bias against disruption of domains must be a consequence of the recombination event *per se*.

**Conclusions/Significance:**

This is the first systematic study relating the units of LGT to structural features at the protein level. Many genes have been interrupted by recombination following inter-lineage genetic transfer, during which the regions within these genes that encode protein domains have not been preferentially preserved intact. Protein domains are units of function, but domons are not modules of transfer and recombination. Our results demonstrate that LGT can remodel even the most functionally conservative modules within genomes.

## Introduction

Genomes are shaped by processes that direct the acquisition and inheritance of genetic material. Since Darwin's *Origin of Species*, vertical (parent-to-offspring) descent within a lineage has been considered to be the main mode of genetic transmission. More recently the role of lateral genetic transfer (LGT) has been emphasized, particularly among prokaryotes [1]–[3], in contributing to the origin of physiological diversity [4]. A transfer event involves the acquisition of an external genetic fragment into the cell and its subsequent integration into the host genome through recombination. These recombined regions might correspond to fragments of genes [5]–[8], intact genes, multi-gene clusters [9], operons, plasmids, or even entire chromosomes [10]. Methods based on molecular phylogenetics normally focus on gene or protein families as the unit of analysis. For example, previous studies that explored the frequency and impact of LGT in prokaryotes at a multi-genome scale [*e.g.* 11]–[18] have been based, explicitly or implicitly, on the assumption that whole genes are the unit of LGT. None of these studies has taken a comprehensive rigorous approach to characterizing the units of genetic transfer independently of gene boundaries.

Genomes of prokaryotes consist largely of protein-coding sequences separated by short intergenic regions. The corresponding proteins often consist of one or more spatially compact structural units known as *domains* which may fold autonomously and which, singly or in combination, confer the protein's specific functions [19], [20]. As natural selection is based on function, we hypothesize that domains might also serve as units of genetic transfer, *i.e.* that regions of DNA that are transferred and recombined might encode intact structural domains of proteins. In support of this view, recombination events that disrupt the folds of bacterial β-lactamases [21] and begomoviral proteins [22] appear to be selectively disadvantageous. We showed earlier, by phylogenetic analysis of 22,437 putatively orthologous protein sets of 144 fully sequenced prokaryote genomes [11], that LGT has contributed significantly to the composition of some genomes. Comparison between the phylogeny inferred for each protein set and a reference supertree (the inferred organismal phylogeny) implied that about 13.4% of the tested relationships (bipartitions) were topologically discordant at a posterior probability threshold of 95% or greater, and had possibly been affected by LGT. In that study, we followed established practice in treating individual proteins (genes) as the unit of analysis.

The dataset developed for that study provides a unique platform to examine the distribution of recombination breakpoints that occur within protein-coding sequences, and the extent to which domain-encoding sequences have been disrupted by LGT. Our three null hypotheses are that (a) recombination breakpoints are uniformly distributed among protein-coding sequences, such that across putatively orthologous gene sets, no correlation exists between the occurrence of recombination breakpoints and the presence of protein structural domains; (b) within gene sets that have annotated domains, recombination breakpoints are uniformly distributed, such that no correlation exists between their location and domain-encoding regions; and (c) breakpoints that fall within domain-encoding regions do not preferentially associate with any particular feature of that region, for example its midpoint or boundaries. Translated to the protein level, the last hypothesis, if true, would mean that protein domains tend neither to be preserved as intact units of genetic transfer, nor to suffer preferential disruption within their core structural region.

To facilitate our presentation of these hypotheses and description of test results, we introduce two new terms: *domon*, a gene (exon) region that encodes a protein domain, and *nomon*, a gene (exon) region that encodes a part of a protein not recognized as a domain. Domon boundaries in DNA thus correspond to domain boundaries in the protein product.

## Results and Discussion

**Figure 1:**
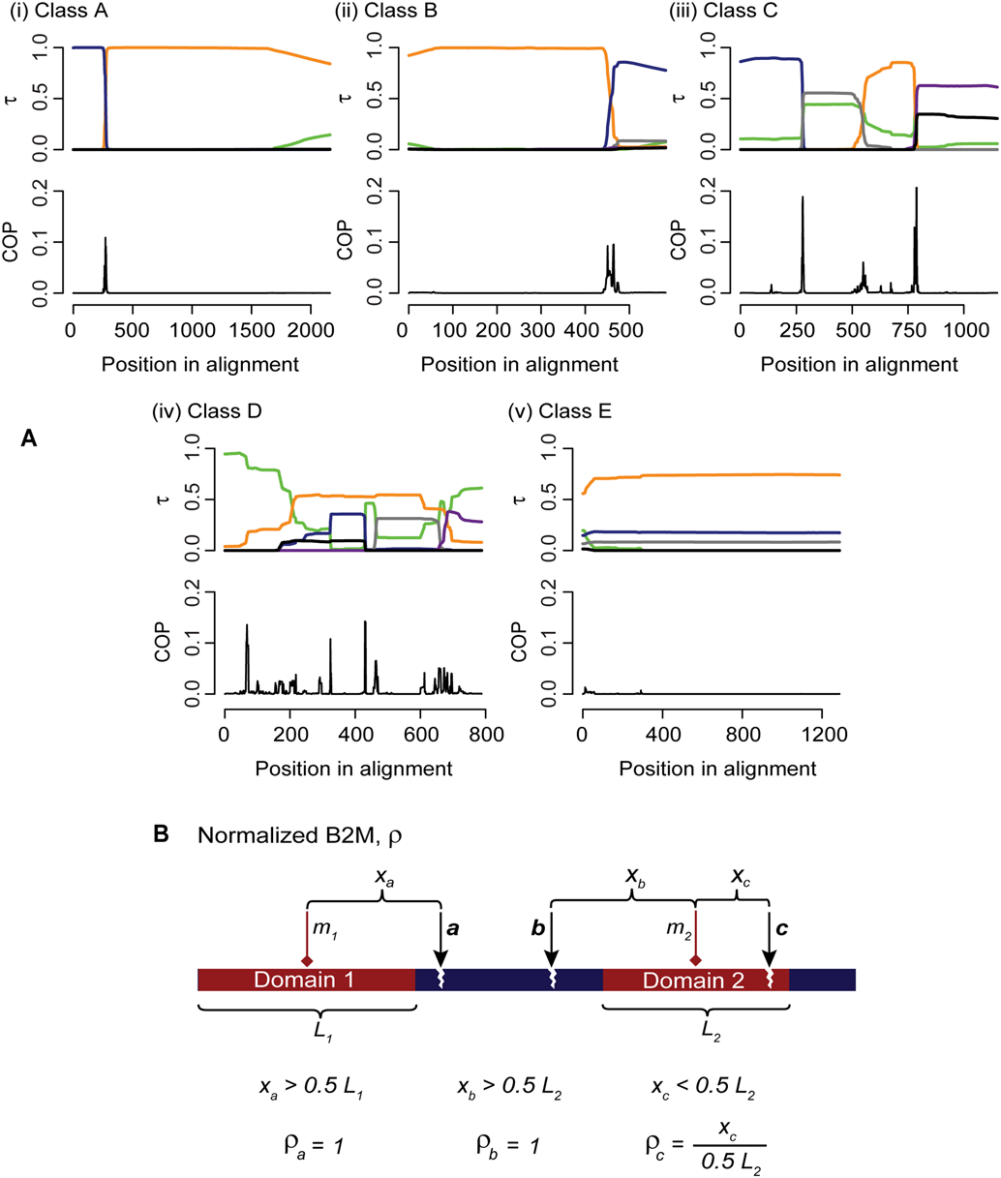
Inference of recombination breakpoints with respect to annotated protein domains. (A) Classification of results in breakpoint identification based on outputs of DualBrothers [25]. In each panel the X-axis represents the positions in the sequence alignment, the Y-axis of the upper graph shows the Bayesian posterior probability (*τ*) of the inferred tree topology, and each colored line indicates a distinct topology (five major topologies are shown; the green line represents the sum of remaining tree topologies). The Y-axis of the lower graph in each panel shows the marginal posterior probability that an alignment position (column) is a breakpoint. Examples are shown for each of classes A–E. Classes A–C present clear evidence of recombination, Class D contains inconclusive cases, and Class E consists of cases for which we find no evidence of recombination. (B) Definition of *ρ*. A protein sequence is illustrated with two predicted domains. The midpoint of each domain is represented by a red diamond (*m_1_* and *m_2_*). Three breakpoints (*a–c*) are illustrated as black arrows. The *ρ* denotes the number of amino acids between an observed breakpoint and the midpoint of the nearest domain, divided by the half-length of the corresponding domain (0.5 *L*), with *ρ_max_* = 1 (where the breakpoint is located at or outside the domon boundary in an aligned gene-sequence set).

To minimize potential confounding effects of duplicated genes, we extracted the 1,462 aligned sequence sets for which no gene is duplicated within the corresponding genome (*i.e.* putatively orthologous gene sets); the number of sequences in each set ranged from 4 to 52. We implemented a two-phase strategy [23] to detect recombination events within these sequence sets. We first applied three statistical methods [24] to detect recombined regions; then in those sequence sets within which a recombined region was detected, we located recombination breakpoints using a rigorous Bayesian phylogenetic approach [25] that infers changes in tree topologies and evolutionary rates across sites within each set (Methods S1 and Figure S1). The Bayesian approach has been shown to perform with high accuracy in locating breakpoints on simulated data [26], but is too computationally demanding for initial genome-wide screening. In this way we classified the 1,462 sequence sets into five categories based on support for alternative topologies and on width (number of alignment positions) of the transition between topologies (Table 1 and Figure 1A). Sequence sets presenting clear evidence of recombination within the gene boundaries were categorized into Classes A (1.6%), B (9.3%) and C (8.6% of the 1,462 sets), with Class A showing abrupt changes in Bayesian posterior probability (BPP) support for alternative topologies in the breakpoint region indicative of recent transfer, Class B showing a more-gradual change in such BPP indicative of a less-recent transfer, and Class C exhibiting a combination of abrupt and gradual changes in BPP. Sequence sets with inconclusive evidence (BPP<0.50 for an alternative topology) or uninterpretably complex patterns were grouped as Class D (5.5%), and those with no evidence of within-gene recombination as Class E (75.0%).

**Table 1:** Classification of results in breakpoint identification.

Classes	A	B	C	D	E
Support (BPP) of alternative tree topologies in breakpoint region	≥0.90	≥0.50	≥0.50	<0.50	N/A
Region length (nt) over which BPP change occurs	1–30	>30	>1	>1	0
Inference of recombination	+	+	+	−	−

The criteria used in the classification are BPP support for alternative tree topologies in the breakpoint region, and number of aligned nucleotide positions (nt) over which the topology changes. Cases in which all breakpoints show abrupt change between very strongly supported topologies constitute Class A, and those in which all breakpoints show more-gradual change between moderately to strongly supported topologies constitute Class B. Class C groups individual cases showing a combination of abrupt and more-gradual BPP changes across breakpoints. Classes A–C represent positively identified recombination events, and precise breakpoints were inferred. Cases showing inconclusive support (BPP<0.50) at breakpoint regions, or uninterpretably complex patterns, were assigned to Class D, and those that show no change were classified as Class E. ‘N/A’ denotes *not applicable*.

Correlation between the identified recombination breakpoints and position of protein structural domains was investigated using the Structural Classification of Proteins (SCOP) database [27]. Among the 657 sequences with domain annotation (Table S1), 497 (75.6%) are annotated as having a single domain, 138 (21.0%) two domains, and the remaining 22 (3.3%) three or more domains; 111 sequences (16.9%) are annotated as all-inclusive domains (>99% of residues within one or more domains). In total, 861 domains are annotated, covering 6.34% of the amino acid residues and 6.69% of the protein sequence alignment columns in these single-copy protein sets.

### Over-representation of recombination breakpoints in gene sets with domain annotations

Of the 1,462 gene sets, 286 (19.6%) exhibit clear evidence of recombination (Classes A–C). In all, 820 recombination breakpoints were identified within these sets, yielding an average of 2.87 breakpoints per gene set. Of the 1,462 gene sets, 81 (5.5%) have annotated domain information (one or more domains annotated in one or more protein sequences from each set) and 48 of these (59% of 81, 17% of 286) were inferred to have at least one recombination breakpoint. These 48 contain 166 breakpoints, 20.2% of the total 820. Thus recombination breakpoints are 3.27-fold over-represented in gene sets with annotated domains (and domains are similarly over-represented in gene sets with breakpoints). Even if the sequences examined in this study are under-annotated with SCOP domains, the over-representation of recombination breakpoints in gene sets with domain annotations suggests that breakpoints do not occur uniformly at random in genes, but instead are preferentially associated with genes that encode protein domains (or with genomic regions close to encoded protein domains). Indeed, a one-sided binomial test (*x* = 48, *n* = 81, *p* = 286/1462) strongly rejects chance (*p*<10^−14^) as an explanation for the over-representation of these breakpoints in gene sets with annotated domains. Thus breakpoints are not uniformly distributed throughout protein-coding sequences, and our first null hypothesis can be rejected.

### Do recombination breakpoints preferentially avoid domons?

Our second null hypothesis states that recombination breakpoints are uniformly distributed within domon-containing gene sets, such that no correlation exists between breakpoints and the locations of domons. To test this, we focus on the 48 gene sets that contain at least one clear recombination breakpoint (Class A–C), and also encode at least one annotated SCOP domain. First we associate each breakpoint uniquely with a domon boundary: if the breakpoint falls within a domon, we associate it with the closer boundary of that domon; and if it falls outside any domon, we associate it with the closest domon boundary in that aligned gene set. We introduce the normalized breakpoint-to-midpoint distance statistic *ρ*, in which distance is assessed as the number of aligned amino acid positions between an inferred breakpoint and the midpoint of the corresponding domain (Figure 1B); where the associated breakpoint lies outside the domon (but within the analysed sequence), *ρ* = 1 by definition. A *ρ* value is observed for each inferred breakpoint in an alignment, so long as that breakpoint is associated with a domon boundary (and hence with a domon) annotated in at least one of the aligned sequences. Where the associated domon varies in length within an alignment (*e.g.* due to insertion or deletion of codons), we nonetheless compute a single *ρ* value for that breakpoint, with its value calculated as the average of the individual breakpoint-to-midpoint values for each sequence. A large *ρ* value (*ρ*≈1) indicates that the associated breakpoint is located far from a domon midpoint (*i.e.* close to or beyond the domon boundary), and thus that the protein domain has remained structurally intact, or mostly so, during recombination. In contrast, a small *ρ* value (*ρ*≈0) indicates that the associated breakpoint is positioned close to a domon midpoint, and thus that the core of the corresponding protein domain is likely to have been disrupted by recombination.

**Figure 2:**
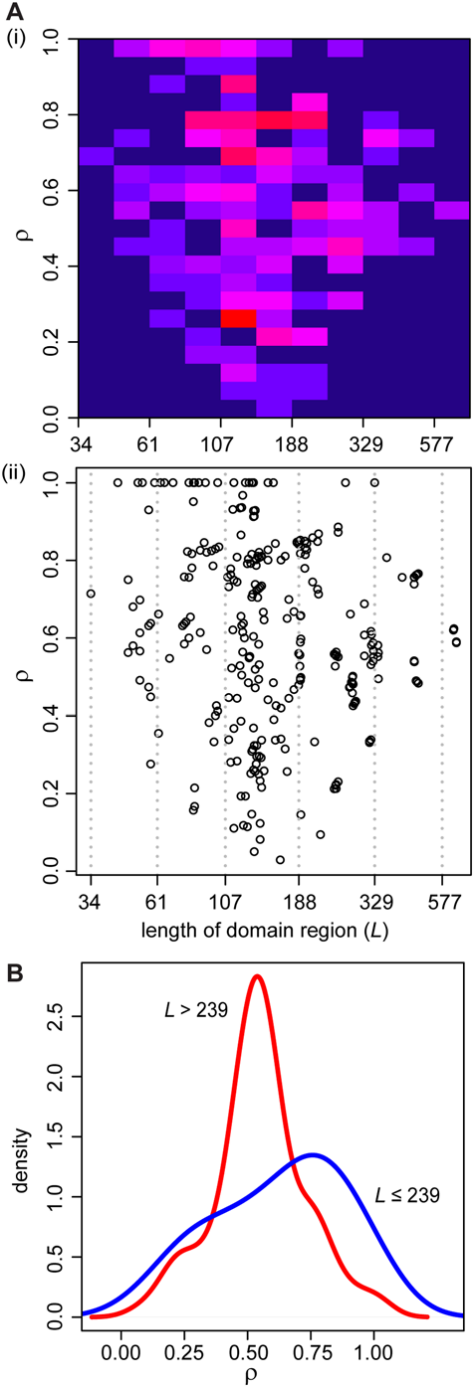
Relationship between *ρ* and domain lengths. (A) Relationship between *ρ* and the length of the corresponding domain region for each inferred breakpoints in the dataset shown as (i) a heat map and (ii) a dot plot (*n* = 311, including *ρ* = 1). The color in each cell in the heat map ranges from dark blue (the fewest data points, minimum 0) to bright red (the most data points, maximum 13). The column of domain lengths 105–142 contains the most data points, 92. Domain lengths in the X-axis for both (i) and (ii) are shown in natural logarithmic scale. (B) Density plot of *ρ* for instances in which the corresponding domain length, *L*≤239 (blue line, *n* = 242) and instances in which *L*>239 (red line, *n* = 69). The *ρ* distances in cases in which *L*≤239 are significantly greater than those where *L*>239.

**Figure 3:**
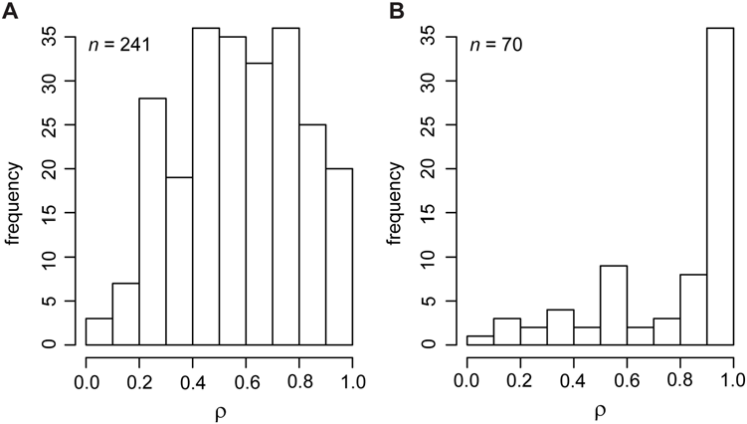
Relationship between *ρ* and domon coverage of the genes. Two instances are shown, when domon coverage on the aligned gene sequence is (A) greater than or equal to 80%, or (B) less than 80%, of the total sequence length. The sample size in each distribution (*n*) is shown in the top left corner of the panel. Large *ρ* values≈1 suggest that the respective recombination breakpoint locates at or outside the domon boundary, avoiding disruption of the domon region.

We examined the relationship between *ρ* and the length of the corresponding domons, including breakpoints outside domons, as shown in Figure 2A. Of the 311 *ρ* values inferred in the dataset, many (*n* = 92, 29.6%) associate with a protein domain of length 105–142 residues. One striking observation from Figure 2A is the enrichment of *ρ*≈1 in cases where the corresponding domain region length is ≤239 residues; this is not observed where the corresponding domain region length is >239 residues. To illustrate this more clearly, Figure 2B shows the density distributions of *ρ* for these two instances. A Kolmogorov-Smirnov test between the two distributions yielded *D* = 0.38 and *p*<10^−7^, strongly suggesting that they differ significantly from each other. There is thus a strong bias for breakpoints associated with shorter domons to be located relatively far from the domon midpoint or outside the domon, *i.e.* small protein domains tend to be largely or wholly conserved in recombination. In contrast, breakpoints associated with longer domons are not similarly biased to avoid the midpoint, *i.e.* larger protein domains tend to be disrupted by recombination. This bias could result, at least in part, from the interplay between the lengths of domons and non-domon regions (nomons), as shown in Figure 3. As the proportion of nucleotide positions in domons becomes large (≥80% of the aligned gene length: Figure 3A), it becomes correspondingly less likely that randomly located breakpoints will fall outside domons (*i.e.* inside nomons), hence harder for *ρ* to attain its maximum value (bounded by definition at 1). When the proportion of nucleotides in domons is small (<80%: Figure 3B), there is a better chance that a breakpoint can locate outside the domon compared to the former cases, and we observe *ρ*≈1 (non-uniform distribution at *p*<0.05), suggesting that breakpoints are avoiding these domons. An alternative, if at this point speculative, explanation might be that large domains tend to consist of smaller structural features (sub-domains) of functional significance and therefore selective value, but these sub-domains are distributed irregularly within large domains. Devising a test to distinguish between these two alternative explanations poses an interesting challenge in computational structural biology.

### Are domains preserved intact during recombination?

**Figure 4:**
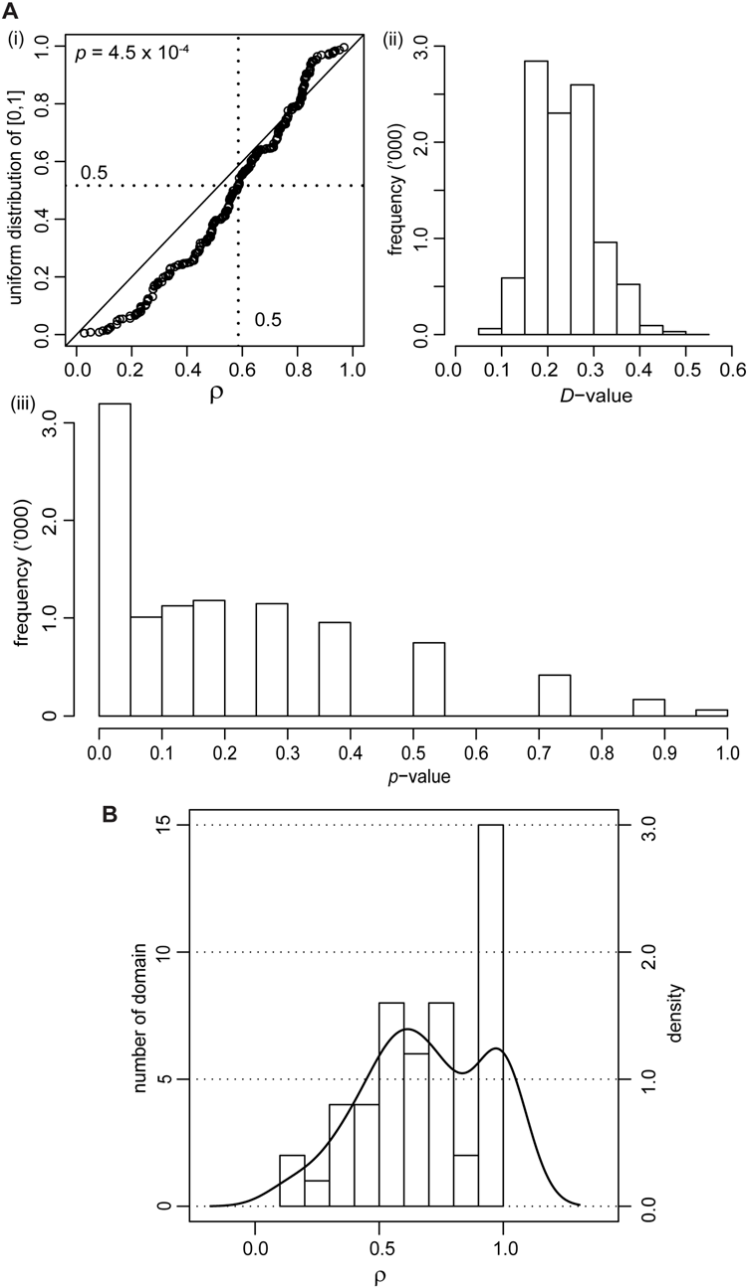
Distribution of *ρ*. (A) Panel (i) shows a quantile-quantile plot between all *ρ* from domain-associated breakpoints and the uniform distribution on [0,1]; *n* = 286, excluding *ρ* = 1. If the distribution of *ρ* is identical to uniform, points in the quantile-quantile plot would follow the diagonal line from (0,0) to (1,1). The dotted lines indicate quantile = 0.5 on both axes. A Kolmogorov-Smirnov test was used to evaluate whether the difference between these two distributions was significant, yielding *D* and *p* values. Panels (ii) and (iii) show the distributions of *D* and *p* values that resulted from Komogorov-Smirnov tests between each of the 10,000 subsamples of the dataset and the respective uniform distribution [0,1]. (B) Distribution of *ρ* across distinctive domains, as histograms (bars) and density plot (line). Details of these domains are listed in Table S2.

Recombination breakpoints that fall within domain-encoding regions (domons) are expected to be distributed uniformly-at-random therein. If so, recombination would neither preferentially preserve, nor avoid disruption of, core domain structure, for which we use the domon midpoint as proxy. The analysis described in the previous section was designed within the context of whole gene sequences; here, we focus on domons themselves. To test this hypothesis, we constructed a quantile-quantile plot to compare the observed distribution of *ρ* values to the null (uniform) distribution on [0,1] (Figure 4A). Since a breakpoint that lies outside a domon (but within the analyzed sequence) is assigned *ρ* = 1 by the definition of our normalized scoring strategy (see Figure 1B), we omit these instances in this part of analysis. If the distribution of sequence breakpoints shows no correlation with any particular region of the domon as expected under our null hypothesis, the *ρ* values are expected not to deviate significantly from the uniform distribution. To adjust for inference bias due to large sample size (which yields artificially small *p*-values), we sub-sampled the dataset randomly (50 samples, 10,000 times) and compared each sub-sample to a uniform distribution on [0,1] using a Kolmogorov-Smirnov test, yielding distributions of the 10,000 *D* test statistics and *p* values. The *D* statistic represents the magnitude of difference between the two distributions, and *p* indicates the significance of this observed difference; both range between 0 and 1. We observed small values of *D* (mean 0.24), indicating that the deviation of each subsample from the uniform distribution is small, and moderate values of *p* (mean 0.2, with 32% of the *p* values<0.05), suggesting that this deviation from uniformity is at best only marginally significant (Figure 4A). Therefore, recombination breakpoints that fall within a domon show little or no tendency to localize away from its center, *i.e.* recombination breakpoints do not avoid disrupting core protein-domain structure, and under this test we find no compelling evidence to reject our third null hypothesis. See Methods S1 and Figure S2 for more information about the percent identity of the observed domon and nomon regions in the dataset. The lengths of domain regions and their relationship with the lengths of inter-domain regions across the dataset are shown in Figure S3 and Figure S4 (see Methods S1 for details).

### Types of protein domains

We found boundaries corresponding to a total of 50 distinct types of protein domains to have an associated *ρ* value. We pooled these *ρ* values based on the individual domain description in SCOP (irrespective of length variations) to examine whether certain types of protein domains tend to be conserved or disrupted in the event of recombination. These protein domains and their respective average *ρ* values are listed in Table S2, and their distribution is shown in Figure 4B. Fully 30% of these domains have an associated *ρ* between 0.9 and 1.0, and for 13 (26%) of these *ρ* = 1. The *ρ* values are not uniformly distributed (Kolmogorov-Smirnov test *D* = 0.43, *p* = 10^−3^). While our observations suggest that certain domain types tend to be conserved or disrupted in the event of recombination and that this observation is unlikely to be explained by chance, a greater number of domain types will need to be sampled for this hypothesis to be tested with the necessary rigor.

### Selection versus recombination

Our observations raise the question of whether the bias in breakpoint location results from selection against nucleotide substitution on one side of the domon boundary and accumulation of substitutions on the other, or is alternatively a consequence of the recombination event *per se*. The cases of recent transfer into the gene (Class A breakpoints) cannot be explained by selection: the abrupt change in BPP support for alternative topologies in the breakpoint region indicates a recent event, leaving insufficient time for substitutions to accumulate. For cases of less-recent transfer (Classes B and C), invoking selection on substitution processes to explain the more-gradual change of BPP would imply the existence of substantially different substitution rates between introgressed and background sequences; this is not the case, as using a Bayesian approach [25] we found no instance among the alignments in Classes B and C in which substitution rates differ by more than 0.30 substitutions per site across the entire alignment. Nor can the proximity of breakpoints to domon boundaries be attributed to the truncation (or extension) of domains, as the mean length of homologous domains is not significantly different (*p*-value = 0.48) in the presence of recombination (182 amino acid residues) compared to its absence (173 amino acid residues).

Genomes evolve in modular fashion, with different evolutionary histories for different regions [1], [3]. Our work shows that LGT among distantly related taxa, or at least the component of homologous recombination that mediates the introgression of such genetic material into the host chromosome, can produce genes with mosaic ancestries. In other words, the units of genetic transfer are not restricted to whole genes [5]–[9], consistent with the relatively small recombination fragment sizes found in some [28] but not all [28], [29] species. Breakpoints of within-gene recombination exhibit a strong association with sequences containing annotated protein domains (and hence domons), and large domains generally have not remained intact during and/or after LGT. Other LGT may transfer entire genes or groups of genes [*e.g.* 10], although these cases are not detected by the methods we applied here. Our findings suggest that fixation of transferred genetic fragments in bacterial populations does not correlate with forces of natural selection that are expected to maintain intact protein domains.

## Materials and Methods

### Dataset

From 144 completely sequenced prokaryote genomes in a previous work [11] we identified 22,437 putatively orthologous protein sets of size *N*≥4 using a hybrid clustering approach [30]. We aligned these sequence sets and validated the alignments using a pattern-centric objective function [31]. The resulting amino acid alignments were then computationally reverse-translated to nucleotide alignments using the corresponding nucleotide sequences from GenBank (http://www.ncbi.nlm.nih.gov/), with the arrangement of the nucleotide triplets reflecting the protein alignment in each case (gene set). To minimize erroneous inference arising from the presence of paralogous sequences within these sets, we further restricted our dataset to those 1,462 sequence sets for which each member represents a different genome. These sets of single-copy genes range in size from 4 to 52 members each, and total 11,128 sequences. The pairwise nucleotide identity across all sequences in each set is roughly normally distributed around mean 52.8% (minimum mean identity 35.3%, maximum mean identity 93.9%, standard deviation 8%).

### Detection of recombination

We adopted a two-phase strategy for detecting recombination in nucleotide sequences [23]. During the first phase, we used three statistical measures [24] to detect occurrences of recombination based on discrepancies of phylogenetic signals across the sequence set at the nucleotide level. In sets in which at least two of the three tests are positive for the presence of recombination, we subsequently employed a rigorous Bayesian phylogenetic approach [25] to more-accurately locate recombination breakpoints. The implementation of this strategy is described in detail in the Methods S1.

### Annotation of protein domains

Protein domain and boundary information for each of the 11,128 proteins in the dataset was determined by sequence similarity search against domain entries in Structural Classifications of Proteins (SCOP) version 1.69 [32], at the *e*-value cut-off of 10^−3^.

## Supporting Information

Methods S1 Methods in detail.
(0.15 MB PDF)

Table S1 Number of gene sets (alignments), sequences, annotated domains and the inferred recombination breakpoints in this study. N/A denotes not applicable.
(0.01 MB PDF)

Table S2 List of distinctive protein domains and each respective average *ρ* value of its associated breakpoints inferred in this study. Large associated *ρ* values (*ρ*≈1) indicate these domains tend to be conserved, whereas small associated *ρ* values (*ρ*≈0) indicate that these domains tend to be disrupted in the event of recombination.
(0.10 MB PDF)

Figure S1 Identification of a recombination breakpoint based on change-of-profile (COP) profile plot from DualBrothers. The Y-axis represents the marginal posterior probability of the position in the alignment being a COP, while the X-axis represents the positions in the sequence alignment. The breakpoint was defined as the median of the sample distribution. The shaded area represents the area bounded within the 95% Bayesian Confidence Interval, as identified between quantiles 0.025 and 0.975.
(0.15 MB TIF)

Figure S2 Sequence identity within the dataset. (A) Sequence identity across the whole dataset, within domon (X-axis) and within nomon (Y-axis) regions, based on SCOP annotations. The trend-line describing the linear relationship between the two axes is shown. The two distributions differ very little from each other (*D* value 0.16 in Kolmogorov-Smirnov test) although the difference may be statistically significant (*p* value 0.008). (B) Distribution of the ratio of percent identity within domon and within nomon regions (D/N ratio) in the dataset.
(0.25 MB TIF)

Figure S3 Distribution of the lengths of domain regions in the dataset.
(0.14 MB TIF)

Figure S4 Relationship of the length of domain region (X-axis) and that of inter-domain region (Y-axis), shown for sequences in which recombination is inferred. The relationship is shown as (A) a heat map and (B) a dot plot. In the heat map, blue cells contain the least number of data points (minimum 0), while the bright red contain the most number of data points (maximum 13). Both X and Y axes are shown in natural logarithmic scale.
(0.46 MB TIF)
