# Supplementary material for: Are Protein Domains Modules of Lateral Genetic Transfer?
Source: PLoS One. 2009 Feb 20;4(2):e4524. doi: 10.1371/journal.pone.0004524 (PMC2639706; doi:10.1371/journal.pone.0004524)
Supplement: Methods S1 — Methods in detail. (0.15 MB PDF) [file pone.0004524.s001.pdf]

Supporting Information

Methods S1

Two-phase strategy for detecting within-gene recombination [1]

Phase I: Screening for instances of recombination

We used three quick and simple statistical tests to screen the 1,462 gene-sequence sets for instances of recombination: (a) maximal chi-squared (MaxChi) [2], (b) neighbor similarity score (NSS) [3], and (c) pairwise homoplasy index (PHI) [4]. These three tests measure the significance of phylogenetic discrepancy across sites in an alignment, and are implemented in PhiPack [4].

(a) Maximal chi-squared test (MaxChi) [2]

Within each sequence set, a sliding window is applied on each possible pair of sequences. The chi-squared statistic is computed to compare the proportion of identical sites within the left half-window with the proportion of identical sites within the right half-window. Recombination is likely when there is a significant discrepancy between the two proportions. Therefore, the maximal chi-squared over all sequence pairs is inferred as evidence of recombination. The fixed window size used in the test was set to equal two-thirds of the number of polymorphic sites in the sequences, as recommended in previous studies [5,6].

(b) Neighbor similarity score (NSS) [3]

Recombination is likely to have occurred when the aligned sequence set contains a cluster of topology-informative sites (columns) that indicate a phylogeny topologically incongruent with the phylogeny indicated by the other sites within that alignment. The NSS statistic is a measure

of the extent and the significance of such clustering. Intuitively, when pairwise comparisons of all informative sites are laid out in a compatibility matrix and compatibility and incompatibility of sites are color-coded, the NSS is simply the fraction of adjacent squares of the same color in the matrix.

(c) Pairwise homoplasy index (PHI) [4]

In sequence comparison, homoplasy is an observation of same or similar character states that do not share a common ancestral origin. A recombination event would create a homoplasious region in the sequence. Similar to NSS, PHI is a compatibility-based statistic that compares topology-informative sites in a pairwise matrix. The main difference between the two statistics is that NSS measures the clustering of incompatible sites, whereas PHI measures the compatibility between closely linked sites using a refined incompatibility matrix and a refined incompatibility score.

The significance for each of these three statistics was assessed by randomly permuting the columns of the alignment (for MaxChi), or parsimoniously informative sites in the alignment (for NSS and PHI), 1,000 times. We earlier found that biases exist in each of these three tests when detecting reciprocal and non-reciprocal recombination events, and therefore one should not rely on a single test when, as is usually the case, one does not know *a priori* whether an event is reciprocal or non-reciprocal [1]. Here, we accepted that there exists evidence of a recombination event when two of the three statistics had p-values ≤ 0.10. We find that our approach has a 96% specificity in detecting recombinants at the p-value threshold of 0.10 (unpublished). Given the computational efficiency of implementations of these tests, there is no great burden associated with computing p-values for all three, even on thousands of sequence sets.

Phase II: Identification of recombination breakpoints

Once recombination was detected in a gene sequence set, we used a rigorous Bayesian phylogenetic approach, DualBrothers [7], to identify the corresponding recombination breakpoints. We applied a modified version of the program to infer the change-points in phylogenetic signal across the alignment. The Bayesian approach was found to show high accuracy in identifying recombination breakpoints, although the approach itself is computationally expensive in time and memory [8].

DualBrothers is an implementation of reversible-jump Markov chain Monte Carlo (MCMC) to perform inference under a dual multiple change-point model, in which change-points in tree topologies and change-points in evolutionary rates across sites within a sequence set are modeled independently [7]. A prior distribution on the number of change-points creates a strong preference for fewer change-points, which corresponds to the *a priori* assumption that recombination and changes in substitution rate are a rare occurrence. For a given aligned gene sequence set, MCMC was implemented in DualBrothers to sample the joint posterior distribution of phylogenetic trees relating the sequences, the change-points in phylogeny along the alignment, and the change-points in the substitution rate along the alignment. Trees are assumed to be unrooted with exponentially distributed branch lengths. The mean value for the exponential is taken to be the substitution rate, which itself is Poisson distributed. The ratio of nucleotide transititon to transversion (parameter kappa) was also sampled in DualBrothers, emulating an HKY model of nucleotide substitution.

A sequence set of size n has $(2n\text{-}5)!!$ possible unrooted tree topologies relating the taxa. For many of the sequence sets in our dataset the value of n is large, creating an intractable search

space of possible topologies for the DualBrothers MCMC sampler. At large *n*, typically a relatively small number of topologies dominate the posterior distribution and many topologies are extremely unlikely given the data. DualBrothers proposes new updates of topology uniformly-at-random from the set of all possible tree topologies, so unlikely topologies can be proposed, with the consequence that convergence and mixing of the MCMC chain becomes inordinately slow. To concentrate the sampling efforts of DualBrothers on tree topologies likely to be well-represented in the joint posterior distribution, we implemented a preprocessing method suggested by the authors [7] to identify candidate topologies and restrict the search space. We applied MRBAYES [9] version 3.1.2 to sliding windows for each of the sequence alignment. The parameters for MCMC implementation in MRBAYES were set at chain length = 2,500,000 generations, and burn-in = 500,000 generations. For the present work we used a window size of 100 nucleotides and a window-shift value of 25 nucleotides. On an alignment of 1,000 columns, for example, we would run MRBAYES on $\left(\frac{1000-100}{25}\right)=36$ overlapping windows.

From each window we saved the top 90% most-likely trees inferred by MRBAYES (at 90% Bayesian Credible Interval) and pooled the topologies from each window in the sequence set into a list of trees. For windows with little nucleotide diversity, the posterior distribution of topologies can be extremely diffuse because little information exists to resolve branching order for certain taxa. In such scenarios, we further sub-sampled the 90% BCI to arrive at a set of 1,000 candidate trees, with the rationale that the sub-sampled set of 1,000 trees will contain representatives of the major splits present in the posterior, along with arbitrary resolutions of branching order in unresolved portions of the tree.

Finally, we applied DualBrothers to sample recombination breakpoints, restricting the search space to a maximum of 1,000 candidate topologies. MCMC chains were run for 2,500,000 generations with burn-in at 500,000 generations. Markov chain convergence was assessed for 15 arbitrarily chosen gene sequence sets (of various sizes) by running four additional chains and computing the standard deviation of split frequencies as implemented in MRBAYES [9], although on a site-by-site basis instead of on the alignment as a whole. Convergence of Markov chain was not observed in sequence sets of size > 20, even with longer chain runs at 5,000,000 generations. These sets were therefore excluded from the analysis.

In DualBrothers, the marginal posterior probability (mPP) that each position in the alignment is a change-of-point (COP) was calculated. These mPPs, when plotted against positions (columns) in the alignment, form the COP profile plot, on which the identification of breakpoints was based as illustrated in Figure S1. A sharp peak in the COP profile corresponding to a change of tree topology in the sequence set was identified as the breakpoint region. To define the exact location of the breakpoint within a peak, the algorithm searches to the left and right of the peak until the cumulative COP mPPs ≈ 1. This conditional region was treated as an MCMC sample from a discrete-value distribution. A sample distribution was obtained by multiplying the mPPs with the number of MCMC samples in DualBrothers run. The breakpoint location was identified as the median of the sample distribution thus constructed, and the 95% Bayesian Confidence Interval was identified between quantiles 0.025 and 0.975.

Annotation of protein domains

The number of gene sets, annotated protein domains and the inferred recombination breakpoints within the gene sets are shown in Table S1.

Among these 1,462 gene sets, average sequence identity is 0.5647 ± 0.1130 %. Average sequence identity is 0.5335 ± 0.0089 % in the 81 gene sets with domain annotation, and 0.5666 ± 0.1140 % in the 1,381 without domain annotation. A Kolmogorov-Smirnov test comparing the latter two distributions yields $D = 0.1632$ and $p = 0.034$, indicating that the sequence identity levels are only slightly different, but that this difference may be significant. Note that the identity is *lower*, on average, in the sets with domain annotation.

Across the dataset, the average sequence identity within domain-encoding gene regions (*domons*) is 0.52 (minimum 0.36, maximum 0.72, standard deviation 0.085), whereas the average sequence identity within non-domain-encoding gene regions (*nomons*) is 0.47 (minimum 0.22, maximum 0.98, standard deviation 0.12) (Figure S2A). Application of the Kolmogorov-Smirnov test between the two distributions yielded $D = 0.16$ and $p = 0.008$, suggesting that sequence identity levels differ only slightly, although probably significantly, between domon and nomon regions.

Figure S2B shows the distribution of the ratio of nucleotide percent identities within-domon *versus* in nomon regions (D/N ratio). For most of these sequences, the percent identity within domons is essentially indistinguishable from that in the nomon regions (D/N ≈ 1), although we found a few exceptions for which D/N > 2. The DualBrothers algorithm, like most phylogeny-based approaches, may be assumed to perform more accurately in regions of moderate sequence identity than where identity is extremely high or low. Our results allow us to exclude the possibility that, in general, the preferential association of recombination breakpoints we observe in domon regions is an artifact related to greater sequence identity, although this possibility cannot be excluded for a few datasets individually.

Figure S3 shows the distribution of the lengths of domain regions as annotated in the whole dataset using SCOP. Almost half of the domain regions are of lengths 101-150 amino acid residues. As mean domain length increases relative to the length of a sequence, one can imagine that recombination breakpoints are more likely found within-domain than in the inter-domain regions ($\rho < 1$), potentially introducing a bias in the inference of recombination in relation to protein domains.

We examined the relationship between length of annotated domain and inter-domain regions (in amino acid residues) in the sequences in which recombination was inferred. We found that most inter-domain regions are short irrespective of whether the domain-encoding regions are long or short, as depicted by the concentration of data points (pink and red cells) in which lengths of inter-domain regions are short (between 14 and 40 residues) in Figure S4. In the cases of sequences with long domains, the chance of a breakpoint being found outside a domain ($\rho \approx 1$) is low because the length of inter-domain region in these sequences is short. Therefore our inference of breakpoint locations with respect to domain structure might be biased by the lengths of annotated domains in the sequences.

Relating breakpoint locations to corresponding domains

We developed a normalized breakpoint-to-midpoint distance statistic (ρ, as shown in Figure 1B in the main text). A breakpoint with $\rho \approx 0$ is located closer to the midpoint (center) of the corresponding domain than a breakpoint with $\rho \approx 1$. A breakpoint with $\rho = 1$ is located either at the boundary or outside the corresponding domain. Table S2 shows the list of distinctive protein domains and the respective ρ values of the associated breakpoints. A greater number of domain

types will need to be sampled in order to test the hypothesis of the tendency of certain domain types to be conserved or disrupted in the event of recombination.

The relationship between ρ and the corresponding domain length (L) was assessed by a heat map of the total data points. For each border point of the columns (an arbitrarily pre-defined domain length based on the categorization in the heat map), we performed a Kolmogorov-Smirnov test to examine the significance of difference between the distributions of ρ on the left and on the right of that point. Domain length 239 was found to be the separation point at which the two distributions show the most-significant difference (D = 0.38, $p = 10^{-17}$) and on this basis we divided the breakpoints into two distinct groups: those with corresponding domain length ≤ 239, and those with corresponding domain lengths > 239.

References

1. Chan CX, Beiko RG, Ragan MA (2007) A two-phase strategy for detecting recombination in nucleotide sequences. S Afr Comput J 38: 20-27.

2. Maynard Smith J (1992) Analyzing the mosaic structure of genes. J Mol Evol 34: 126-129.

3. Jakobsen IB, Easteal S (1996) A program for calculating and displaying compatibility matrices as an aid in determining reticulate evolution in molecular sequences. CABIOS 12: 291-295.

4. Bruen TC, Philippe H, Bryant D (2006) A simple and robust statistical test for detecting the presence of recombination. Genetics 172: 2665-2681.

5. Posada D (2002) Evaluation of methods for detecting recombination from DNA sequences: empirical data. Mol Biol Evol 19: 708-717.

6. Posada D, Crandall KA (2001) Evaluation of methods for detecting recombination from DNA sequences: computer simulations. Proc Natl Acad Sci U S A 98: 13757-13762.

7. Minin VN, Dorman KS, Fang F, Suchard MA (2005) Dual multiple change-point model leads to more accurate recombination detection. Bioinformatics 21: 3034-3042.

8. Chan CX, Beiko RG, Ragan MA (2006) Detecting recombination in evolving nucleotide sequences. BMC Bioinformatics 7: 412.

9. Huelsenbeck JP, Ronquist F (2001) MRBAYES: Bayesian inference of phylogenetic trees. Bioinformatics 17: 754-755.
