# Supplementary material for: Are Protein Domains Modules of Lateral Genetic Transfer?
Source: PLoS One. 2009 Feb 20;4(2):e4524. doi: 10.1371/journal.pone.0004524 (PMC2639706; doi:10.1371/journal.pone.0004524)
Supplement: Table S1 — Number of gene sets (alignments), sequences, annotated domains and the inferred recombination breakpoints in this study. N/A denotes not applicable. (0.01 MB PDF) [file pone.0004524.s002.pdf]

Supporting Information

Table S1. Number of gene sets (alignments), sequences, annotated domains and the inferred recombination breakpoints in this study. N/A denotes not applicable.

	Gene set alignments	Sequences	Domains	Recombination breakpoints
All alignments (gene sets)	**1,462**	11,128	N/A	N/A
Alignments with domain annotations	**81**	657	861	N/A
Alignments in which breakpoints are found	**286**	2,355	N/A	820
Alignments with domain information in which breakpoints are found	**48**	415	238	166
