# Supplementary material for: Are Protein Domains Modules of Lateral Genetic Transfer?
Source: PLoS One. 2009 Feb 20;4(2):e4524. doi: 10.1371/journal.pone.0004524 (PMC2639706; doi:10.1371/journal.pone.0004524)
Supplement: Table S2 — List of distinctive protein domains and each respective average ρ value of its associated breakpoints inferred in this study. Large associated ρ values (ρ≈1) indicate these domains tend to be conserved, whereas small associated ρ values (ρ≈0) indicate that these domains tend to be disrupted in the event of recombination. (0.10 MB PDF) [file pone.0004524.s003.pdf]

Supporting Information

Table S2. List of distinctive protein domains and each respective average ρ value of its associated breakpoints inferred in this study. Large associated ρ values ($\rho \approx 1$) indicate these domains tend to be conserved, whereas small associated ρ values ($\rho \approx 0$) indicate that these domains tend to be disrupted in the event of recombination.

Domain	Average ρ of associated breakpoints
Alpha/beta-hydrolases	1.0000
Galactose-binding domain-like	1.0000
HAD-like	1.0000
HlyD-like secretion proteins (Pfam 00529)	1.0000
Homeodomain-like	1.0000
Integral outer membrane protein TolC, efflux pump component	1.0000
Invasin/intimin cell-adhesion fragments	1.0000
KaiA/RbsU domain	1.0000
Periplasmic binding protein-like I	1.0000
Periplasmic binding protein-like II	1.0000
PYP-like sensor domain (PAS domain)	1.0000
Stringent starvation protein B, SspB	1.0000
tRNA-binding arm	1.0000
Glyceraldehyde-3-phosphate dehydrogenase-like, C-terminal domain	0.9298
Glutathione S-transferase (GST), C-terminal domain	0.9176
UDP-Glycosyltransferase/glycogen phosphorylase	0.8540
SGNH hydrolase	0.8227
TPR-like	0.7819

PRTase-like	0.7562
Glutathione synthetase ATP-binding domain-like	0.7484
Winged helix DNA-binding domain	0.7470
PurM N-terminal domain-like	0.7428
ARM repeat	0.7423
Zinc beta-ribbon	0.7143
Thioredoxin-like	0.7114
Six-hairpin glycosidases	0.6894
RmlC-like cupins	0.6416
(Trans)glycosidases	0.6334
Ferritin-like	0.6115
Acyl-CoA N-acyltransferases (Nat)	0.6037
PFL-like glycyl radical enzymes	0.6024
E set domains	0.5915
Ribbon-helix-helix	0.5908
FAD/NAD(P)-binding domain	0.5907
S-adenosyl-L-methionine-dependent methyltransferases	0.5752
P-loop containing nucleoside triphosphate hydrolases	0.5473
PRC-barrel domain	0.5471
DNase I-like	0.5208
Beta-carbonic anhydrase, cab	0.5186
Methenyltetrahydromethanopterin cyclohydrolase	0.4831
Subunit III of photosystem I reaction centre, PsaF	0.4600
T4 endonuclease V	0.4464

F420-dependent methylenetetrahydromethanopterin dehydrogenase (MTD)	0.4448
Hypothetical protein Ta1206	0.3996
HybD-like	0.3774
Multiheme cytochromes	0.3333
NAD(P)-binding Rossmann-fold domains	0.3237
Glucocorticoid receptor-like (DNA-binding domain)	0.2758
Nickel-containing superoxide dismutase, NiSOD	0.1593
Protein kinase-like (PK-like)	0.1273
