# Supplementary figures and images for: Are Protein Domains Modules of Lateral Genetic Transfer?
Source: PLoS One. 2009 Feb 20;4(2):e4524. doi: 10.1371/journal.pone.0004524 (PMC2639706; doi:10.1371/journal.pone.0004524)

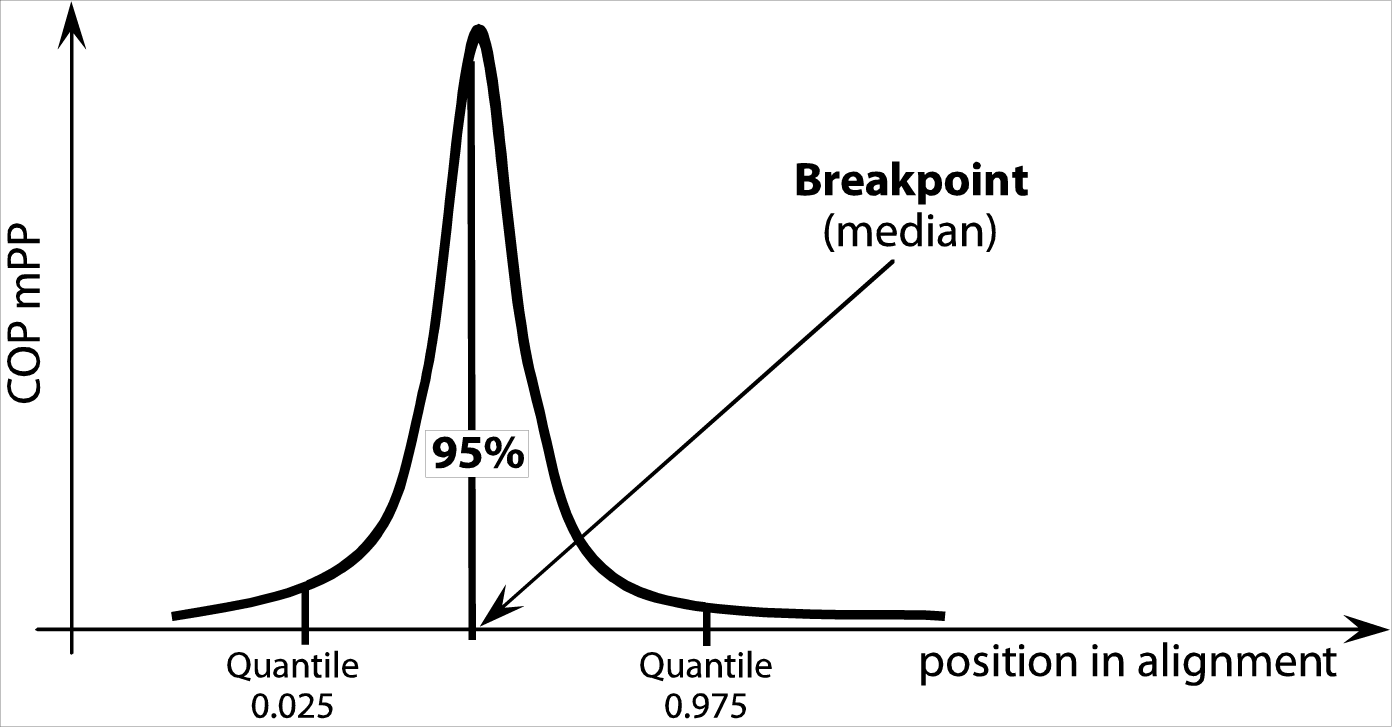

Supplement: Figure S1 — Identification of a recombination breakpoint based on change-of-profile (COP) profile plot from DualBrothers. The Y-axis represents the marginal posterior probability of the position in the alignment being a COP, while the X-axis represents the positions in the sequence alignment. The breakpoint was defined as the median of the sample distribution. The shaded area represents the area bounded within the 95% Bayesian Confidence Interval, as identified between quantiles 0.025 and 0.975. (0.15 MB TIF) [file pone.0004524.s004.tif]

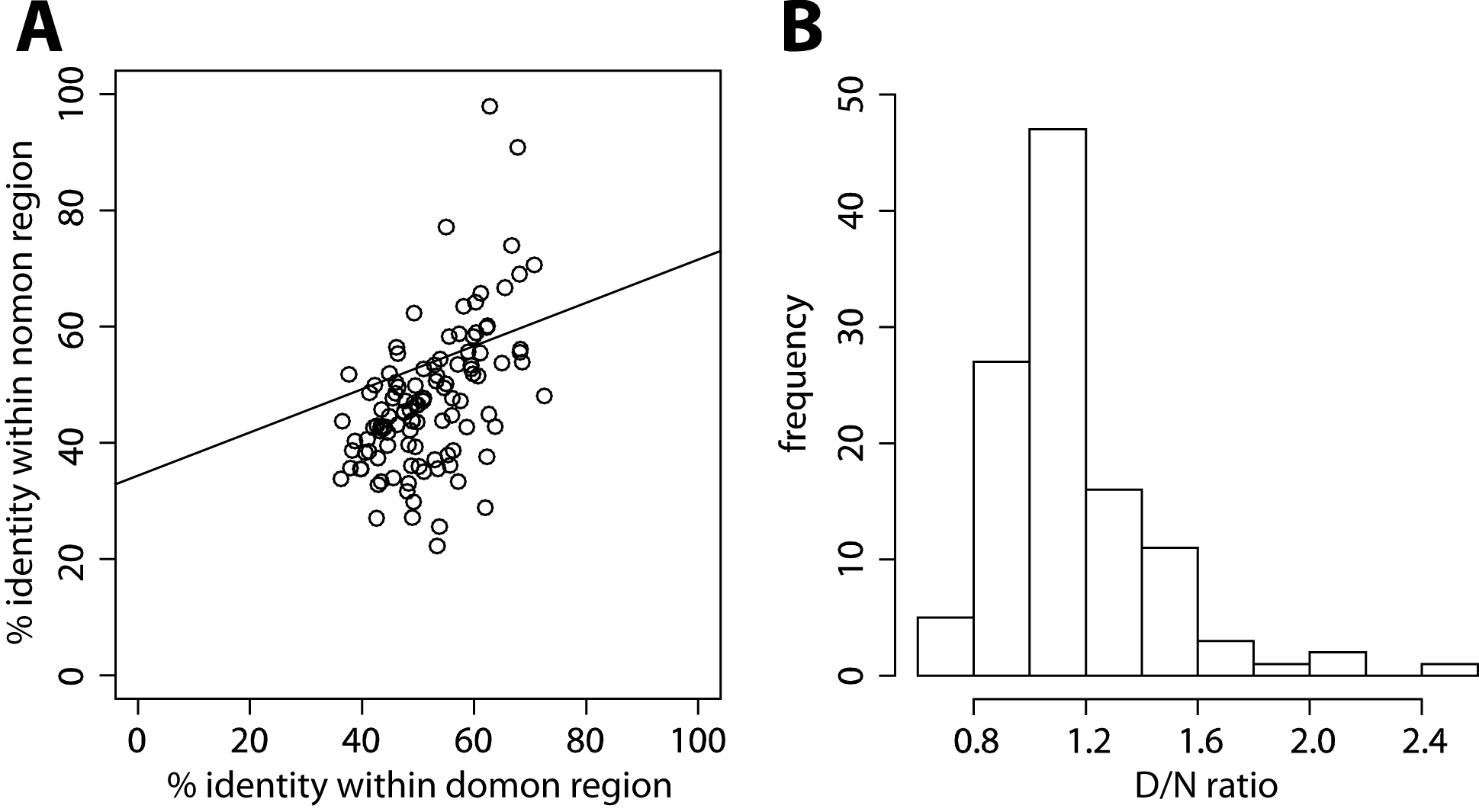

Supplement: Figure S2 — Sequence identity within the dataset. (A) Sequence identity across the whole dataset, within domon (X-axis) and within nomon (Y-axis) regions, based on SCOP annotations. The trend-line describing the linear relationship between the two axes is shown. The two distributions differ very little from each other (D value 0.16 in Kolmogorov-Smirnov test) although the difference may be statistically significant (p value 0.008). (B) Distribution of the ratio of percent identity within domon and within nomon regions (D/N ratio) in the dataset. (0.25 MB TIF) [file pone.0004524.s005.tif]

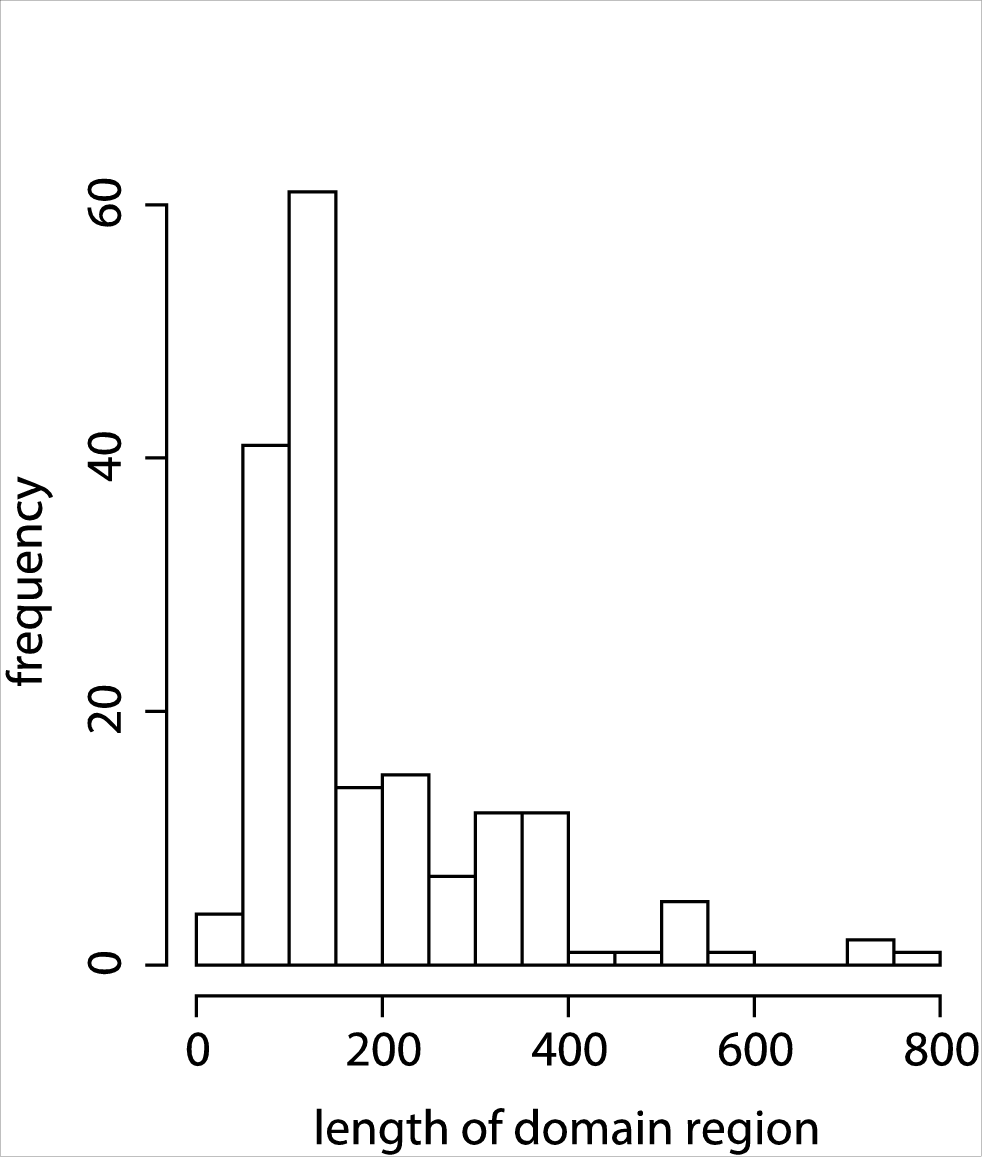

Supplement: Figure S3 — Distribution of the lengths of domain regions in the dataset. (0.14 MB TIF) [file pone.0004524.s006.tif]

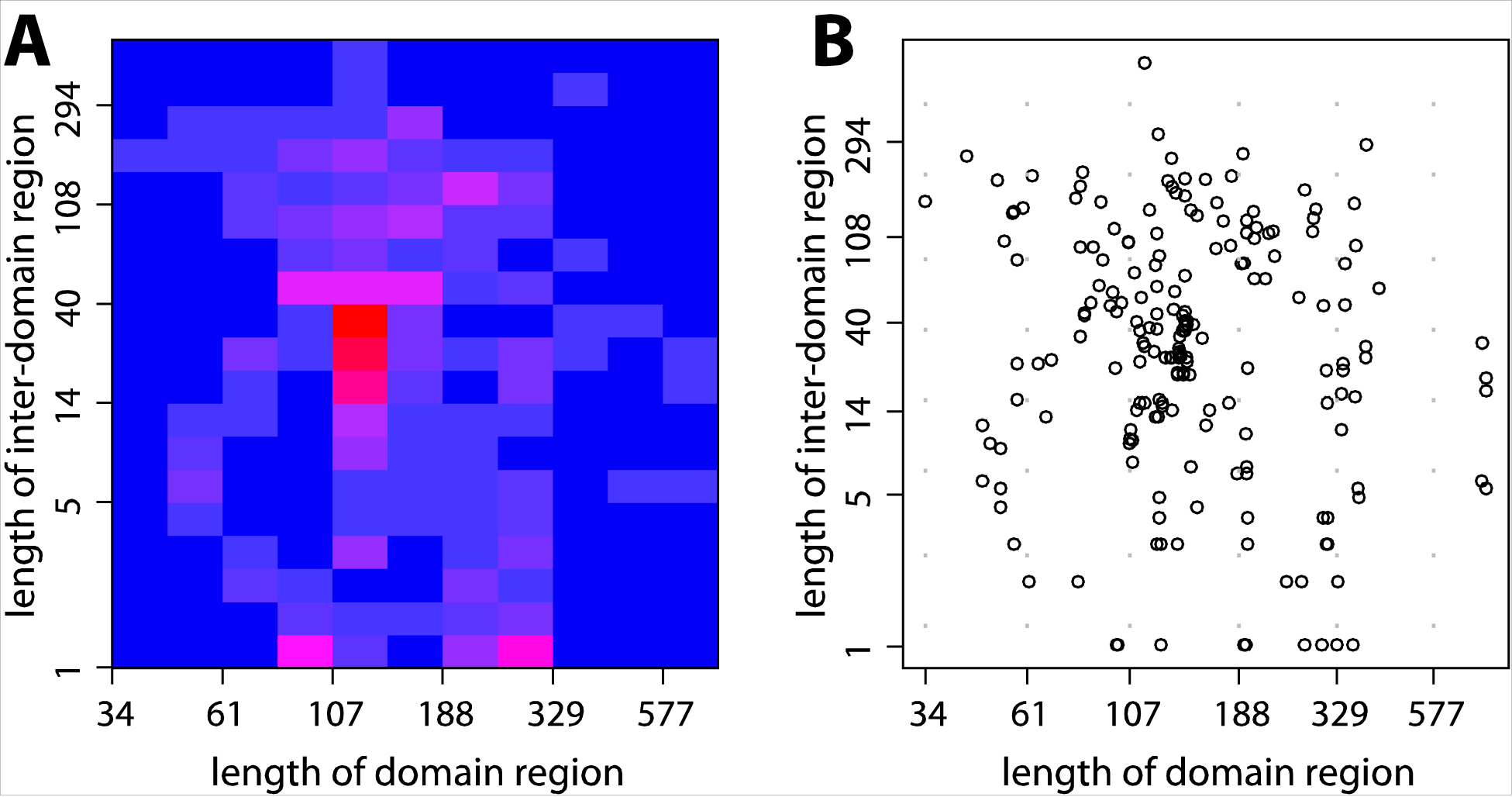

Supplement: Figure S4 — Relationship of the length of domain region (X-axis) and that of inter-domain region (Y-axis), shown for sequences in which recombination is inferred. The relationship is shown as (A) a heat map and (B) a dot plot. In the heat map, blue cells contain the least number of data points (minimum 0), while the bright red contain the most number of data points (maximum 13). Both X and Y axes are shown in natural logarithmic scale. (0.46 MB TIF) [file pone.0004524.s007.tif]
